# Advances in Biomimetic Systems for Molecular Recognition and Biosensing

**DOI:** 10.3390/biomimetics5020020

**Published:** 2020-05-12

**Authors:** Yeşeren Saylan, Özgecan Erdem, Fatih Inci, Adil Denizli

**Affiliations:** 1 Department of Chemistry, Hacettepe University, 06800 Ankara, Turkey; yeseren@hacettepe.edu.tr; 2 Department of Biology, Hacettepe University, 06800 Ankara, Turkey; ozgecanerdem@hacettepe.edu.tr; 3 UNAM-National Nanotechnology Research Center, Bilkent University, 06800 Ankara, Turkey; finci@bilkent.edu.tr; 4 Institute of Materials Science and Nanotechnology, Bilkent University, 06800 Ankara, Turkey

**Keywords:** biosensing, biomimetic, biorecognition, molecularly imprinted systems

## Abstract

Understanding the fundamentals of natural design, structure, and function has pushed the limits of current knowledge and has enabled us to transfer knowledge from the bench to the market as a product. In particular, biomimicry―one of the crucial strategies in this respect―has allowed researchers to tackle major challenges in the disciplines of engineering, biology, physics, materials science, and medicine. It has an enormous impact on these fields with pivotal applications, which are not limited to the applications of biocompatible tooth implants, programmable drug delivery systems, biocompatible tissue scaffolds, organ-on-a-chip systems, wearable platforms, molecularly imprinted polymers (MIPs), and smart biosensors. Among them, MIPs provide a versatile strategy to imitate the procedure of molecular recognition precisely, creating structural fingerprint replicas of molecules for biorecognition studies. Owing to their affordability, easy-to-fabricate/use features, stability, specificity, and multiplexing capabilities, host-guest recognition systems have largely benefitted from the MIP strategy. This review article is structured with four major points: (i) determining the requirement of biomimetic systems and denoting multiple examples in this manner; (ii) introducing the molecular imprinting method and reviewing recent literature to elaborate the power and impact of MIPs on a variety of scientific and industrial fields; (iii) exemplifying the MIP-integrated systems, i.e., chromatographic systems, lab-on-a-chip systems, and sensor systems; and (iv) closing remarks.

## 1. Introduction

Biological, chemical, and physical phenomenological events have always been of interest in various fields of fundamental and applied research. For instance, silk―manufactured by spiders and insect larvae―is spun from a solution, and a water-insoluble material is produced. This solubility variation is attended by conformation (physical) changes of the constituent protein chains, but the configuration (chemical) of the chains remains unmodified [1,2]. A more sophisticated example is the filamentous phage, which is versatile, robust, and tailorable as a promoter of crystalline phase formation [3], and is able to operate the self-assembly of a biomaterial. If the orientation for the self-assembling process is strong enough, the filamentous phage can act as a host that attends to the self-assembly of complex structures [4]. In addition to the examples above, the selective recognition of complex molecules is, in particular, a hallmark of biology, chemistry, and engineering. A brief understanding of natural selection has sharpened the development of several structures, materials, and systems that have been improved for a wide variety of functions [5,6]. Biomimicry, one of the most striking strategies to understand the fundamentals of natural systems, takes lessons from nature’s design and uses this information to create extremely complicated and complex manufacturing systems at different length scales to tackle crucial problems [7].

Over the past few decades, researchers have committed great attention to the design, synthesis, and testing of molecular assemblies that perform recognition and biosensing functions through the fabrication of mimicked structures [8,9,10]. In principle, the designed host molecules can provide similar structural design and features, yet in practice, they have scarcely reached performance levels that compete with biomolecules and permit substrate targeting in a biological fashion [11,12,13,14]. The biomimetic systems comprising models, structures, materials, and strategies mirror the biosynthetic processes toward natural products [15]. Biomimetic systems have a broad range of strategies, which include imitating the nanostructures/architecture of a biological compartment or surface and also mimicking the surface of a bio-receptor that interacts with target molecules. Some of the examples of the first approach are listed as a self-cleaning surface mimicking the nanostructures of lotus flower leaves, and a bone scaffold aiming to mimic bone structure. The selection of a suitable sequence is the gateway for the evolution of biomimetics [16]. The principles of biomimetics can be applied to obtain the functionality of the molecules and techniques to produce the systems [17]. These bioinspired systems are adaptable and inventive, and mimic the natural components of the body [18]. The latter approach, i.e., the bioreceptor-mimicking strategy, aims to replicate the molecular architecture of bioreceptor molecules, opening new avenues in the field of recognition-stemmed platforms, such as sensors, diagnostics, and chromatography. In this context, one of the most crucial bioinspired systems, dubbed molecularly imprinted polymer-based systems, supply a wide range of versatile features, used to imprint target molecules with different molecular weights, sizes, structures, and chemical and physical properties [19,20]. Contrary to the complicated and time-consuming modification techniques, molecular imprinting proposes a sensitive and user-friendly approach for the detection, adsorption, recognition, and separation of molecules in many fields [21,22]. Furthermore, polymers synthesized via the molecularly imprinted method have excellent properties, such as high selectivity, high durability, high stability, reusability, and they are low-cost [23,24,25,26].

In this review, four major points were addressed: the requirement of biomimetic systems, the description and advances of the molecular imprinting method; the recent reports of the molecularly imprinted polymer-integrated systems from the last three years**;** and a statement of closing remarks.

## 2. Why Are Biomimetic Systems Required?

The meaning of the word “biomimetic” comes from the Greek terms “bios” (life) and “mimesis” (to imitate), but its definition is not as easy as the combination of these two words, and it has evolved with technological advances and needs in the field. Because biomimetics is an inspired form of science, specifically a natural entity that employs nature to improve human lives [27], biomimicry can be extensively referred to as a proof of concept, used to accept and adapt nature’s tried-and-tested concepts to tackle challenges [28,29]. Biomimetics also leverage the bar to a higher status by simply applying natural features as the basis to innovate new materials, although these materials can be designed to provide human comfort in the fields of chemistry, biology, physics, architecture, engineering, medicine, and biomedical engineering with examples not limited to the design of gecko-inspired devices to understand adhesion and the self-cleaning mechanisms of geckos, or sketching birds to study and mimic their ability to fly [30,31,32,33,34,35,36].

The shape, texture, motion, and preparation stages of biologically inspired surfaces are largely examined in the invention and evolution of biomimetic systems. In recent years, nanotechnology-based approaches have provided great contributions to the improvement of biomimetic systems, especially biorecognition-stemmed approaches. These systems have provided applications in not only medical applications (cancer treatment, drug delivery, tissue engineering, sensor design, and point-of-care settings), but also environmental applications (water quality, food production, and agriculture) [37,38,39,40,41,42,43,44]. On the other hand, some adversative processes occur in a biological environment that poses some limitations. One of the solutions that could be addressed is a conjugation of passivating agents and targeting the parts on the surface [45]. Molecularly imprinted polymers-integrated biomimetic systems can create actual interactions between the template molecules and functional monomers and can mimic biological recognition [46,47,48].

## 3. Molecular Imprinting Method

The molecular imprinting methods enable specific and selective molecular recognition for desired template molecules [49,50,51]. Basically, three dimensional-biomimetic cavities complementary to the template molecule in shape, size, physical, and chemical functionality can be produced by creating a matrix around the template molecules [52,53]. The polymeric matrix has identical fingerprints of the template molecule as specific cavities followed by the removal of template molecules [54,55,56]. These cavities have identical binding sites and also present outstanding ability for sensitive and specific rebinding of the template molecules through the structure and noncovalent interactions [57,58,59,60].

**Figure 1:**
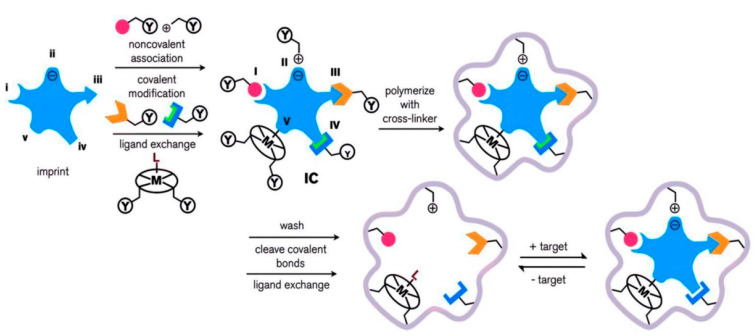
Scheme of the synthesis, and recognition of the molecularly imprinted polymer. Republished with permission from Ansari et al. [61].

As depicted in Figure 1, the molecular imprinting process can be classified into five groups: (i) non-covalent, (ii) electrostatic, (iii) covalent, (iv) semi-covalent, and (v) metal-mediated. An imprinted polymer is associated with a functional monomer, through non-covalent, covalent, or ligand (L) to metal (M) interactions with complementary functional groups. A pre-complex of the template molecule and functional monomer (IC) is formed, in which the functional monomer is bound to the imprinted molecule via (I) hydrogen bonding or van der Waals interactions, (II) electrostatic or ionic interactions, (III) a covalent bond, (IV) a covalent bond with a spacer, or (V) ligand-metal or metal-ligand coordination. The functional group (Y) mixes with a suitable cross-linker. After the polymerization, the imprinted template molecule (target) is removed through washing, cleavage of chemical bonds, or ligand exchange. The imprinted polymeric matrix may also take part in target recognition and binding through non-specific surface interactions that result from surface properties created around the imprinted template molecule during cross-linking [61].

The comparison of different polymerization types in molecular imprinting methods is comprehensively evaluated in Table 1. Up to now, the requirements for polymerization types (including bulk, precipitation, suspension, multi-step swelling, surface, and in-situ) has altered for the preparation of molecularly imprinted polymers [62]. For instance, the bulk polymerization needs the milling of the molecularly imprinted polymers after the polymerization, although irregular-shaped materials can be obtained after the milling and binding sites might be demolished during the pulverization [63]. The precipitation or suspension polymerization types are employed to synthesize a regular-shaped material. The precipitation polymerization is dependent on the growth of polymer chains, which precipitate during the reaction when a specified length of a polymer chain is reached. In suspension polymerization, the reaction occurs in two aqueous and organic phases [64].

## 4. Molecularly Imprinted Polymers-Integrated Systems

### 4.1. Chromatographic Systems

Biomimetic molecular recognition has been applied in chromatographic systems by coupling ligands onto supports through capturing, separating, and determining template molecules. The use of ligands is based on highly specific and reversible interactions that create a selective and effective way [65]. Many different chromatographic systems have been described for determining the desired template molecules. The most common systems include high-performance liquid chromatography (HPLC), gas chromatography-mass spectrometry (GC-MS), spectrophotometry, sodium dodecyl sulfate-polyacrylamide gel electrophoresis (SDS-PAGE), capillary electrophoresis (CE), and electroanalytical methods. More specifically, affinity chromatography is usually applied for protein purification, while the most generally employed adsorbents are natural ligands, such as coenzymes, inhibitors, and antigens [66]. However, these natural ligands have some impediments, including high-cost, poor performance in organic solvents, the need for more sophisticated systems, ligand leakage, and harsh elution condition [67]. On the other hand, molecularly imprinted polymers-integrated systems have high stability at harsh conditions, high performance in organic solvents, high compatible with other technologies, and they are affordable, potentially operated with minimal system requirements, and they could potentially work with any analytes. A special focus is given to molecular imprinting, which is an effective technique to create polymers with specific recognition domains towards template molecules [68].

As an example, Hudson et al. [69] reported an extraction method for antidepressant (fluoxetine) determination in water samples using molecularly imprinted polymers-based chromatographic systems. They successfully synthesized the imprinted polymers using bulk polymerization and optimized with various functional monomers for chromatographic separation. The developed imprinted polymers were employed for the determination of binding capacities in the range of 0–1.5 mM fluoxetine concentration using ultraviolet-visible (UV-Vis) spectroscopy. The researchers also established an HPLC coupled to UV-Vis detection to examine anti-depressant mixtures with other nitrogen-containing compounds. The HPLC analysis showed the preferential binding of the fluoxetine for complex mixtures, potentially pointing out the selectively extract. Overall, the imprinted polymers hold great impact as a promising tool for cleaning water samples, improving aquatic life, and resulting in cost reduction in the pharmaceutical industry at the same time.

As another example, Razym et al. [70] prepared an adsorbent with silica particles using a surface imprinting method and separated Concanavalin A as a representative of the plant protein group (i.e., lectins). In the experimental design, they first modified the surface of silica particles with 3-methacryloyloxypropyltrimethoxysilane, and then synthesized Concanavalin A-imprinted silica particles. In the characterization experiments, these particles were tested with several techniques and determined the parameters on the Concanavalin A adsorption. As a result, they obtained the maximum adsorption capacity as 305.2 mg/g in a wide range of Concanavalin A concentrations (0–2.0 mg/mL) at pH 6.0. They also performed reusability and selectivity studies, and finally verified the one-step purification of Concanavalin A with the surface-imprinted silica particles by performing an SDS-PAGE analysis.

**Figure 2:**
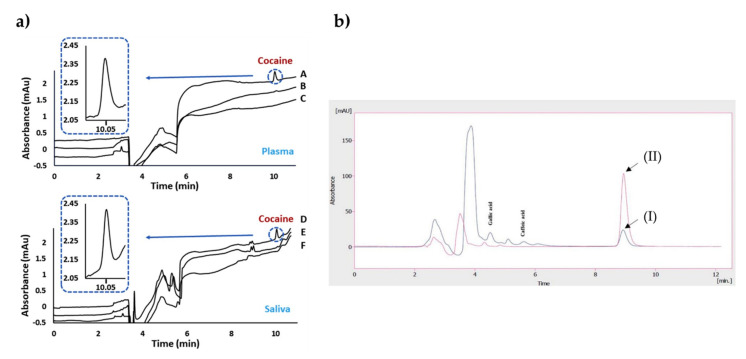
Chromatograms after the extraction of cocaine on plasma and saliva samples (**a**). The indicators in the plot: imprinted (A) and non-imprinted (B) of plasma spiked with cocaine compared to the blank plasma (C); imprinted (D) and non-imprinted (E) of saliva spiked with cocaine compared to the blank saliva (F). Chromatograms before and after extraction of real black tea samples (**b**). The indicators in the plot: unspiked black tea sample before (I) and after (II) extraction. Republished with permission from Bouvarel et al. and Rahimi et al. [71,72].

Bouvarel et al. [71] reported a study on the imprinted monolithic column for the analysis of cocaine in both plasma and saliva samples (Figure 2a). They obtained an extraction recovery values spanning from 85.4% to 98.7% for the plasma samples and also achieved a linear curve between 100 and 2000 ng/mL with a correlation coefficient of 0.999. The researchers performed NanoLC-UV measurements and indicated the selective detection of cocaine in the complex samples after the removal of interfering compounds from the system.

Rahimi et al. [72] developed a sorbent using sol-gel polymerization for solid-phase micro-extraction-based imprinted polymers in order to assign quercetin using the HPLC-UV system. They combined the imprinted polymer and solid-phase micro-extraction methods to increase the stability and selectivity of the fiber. For this purpose, they first modified the stainless-steel wires and prepared the imprinted polymers through the reactions of 3-aminopropyltriethoxysilane and tetraethyl orthosilicate. The researchers then optimized the parameters on extraction efficiency and obtained the maximum efficiency at pH 4.5 while stirring at 500 rpm for 30 min. They obtained chromatograms of real black tea samples before and after extraction (Figure 2b). No interfering peaks were observed in this design. The limit of detection was observed as 9.94 ng/mL. As an outcome, they presented a novel quercetin imprinted solid-phase micro-extraction fiber with impactful thermal and mechanical features on the surface of stainless-steel wire.

In addition, Lu et al. [73] produced dual-template molecularly imprinted polymers by applying precipitation polymerization for norfloxacin and enrofloxacin―crucial target molecules for human and veterinary medicine. They optimized several parameters for obtaining the dispersive solid-phase extraction system that coupled with high-performance liquid chromatography. They isolated high concentrations of norfloxacin and enrofloxacin with linearity between 1.0 μg/L and 200 μg/L with a correlation coefficient above 0.99. The detection and quantification limits were observed 0.22 μg/L and 0.67 μg/L for norfloxacin and 0.36 μg/L and 0.98 μg/L for enrofloxacin, respectively. Subsequently, they validated the dual-template molecularly imprinted polymers-based dispersive solid-phase extraction method by HPLC and tested in real water samples. All the presented work here are also stated in a comparison table (Table 2).

### 4.2. Sensor Systems

Sensor systems integrate a sensing element with a physical transducer such as optical, piezoelectric, or electrochemical whereby the interactions between the target and the recognition molecules are translated into a measurable electrical signal. The sensor systems enable rapid, accurate, labeled/label-free detection while reducing assay time and the need for sample pre-processing steps These systems, therefore, are powerful alternatives to traditional analytical techniques. Furthermore, sensor systems have been integrated with many disciplines, including chemistry, biology, nanotechnology, physics, and electronics. Such integrations have improved the performance of current sensor systems in terms of assay duration, sensitivity, specificity, usability, applicability, and remote monitoring capability. Biorecognition, one of the most crucial elements on sensor systems, has been updated with molecularly imprinted polymers for highly specific recognition and stability, minimizing the current limitations in an antibody of protein-based systems [74]. Sensors combined with molecularly imprinted polymers have been developed for screening purposes in several fields including medical diagnostics, food contamination, and environmental sectors [75,76,77,78,79,80,81,82,83,84].

As an example, Erdem et al. [85] prepared an optical sensor for the detection of *Enterococcus faecalis*, one of the indicators for fecal pollution of water. In the experimental design, they modified the imprinted optical sensor surface using *Enterococcus faecalis*-imprinted nanoparticles that were synthesized via emulsion polymerization. The sensor was tested with different concentrations of bacteria spanning from 2 × 10^4^–1 × 10^8^ cfu/mL, and it was able to detect ~100 bacteria/mL. To assess the specificity and selectivity of the imprinted sensor, they used competitor microorganisms, and the sensor provided high selective and repeatable results within a short period of assay time. Owing to the pivotal properties of imprinted nanoparticles compared to bulk polymers, they presented a fascinating modification method utilizing the template molecule itself, and the sensor surface provided a broad range of versatility to replicate the other template molecules with different molecular structure, size, and physicochemical properties.

Investigation of mycotoxins in agricultural areas and crops necessitates the evolution of highly sensitive and precise methods since the specification of contamination levels even in trace amounts is vital. In the recent literature, a smartphone-based optical sensor decorated with molecularly imprinted polymeric membranes were developed to detect aflatoxin B1 [86]. They used computational modeling for the optimization of the composition of membranes and 2-acrylamido-2-methyl-1-propane sulfonic acid and acrylamide were employed as functional monomers while creating selective binding sites for aflatoxin B1 via in-situ polymerization. In the selectivity experiments, they evaluated the sensor with the other competitor molecules, i.e., aflatoxin G2 and ochratoxin A. The optical sensors provided a high selectivity for the detection of aflatoxin B1. They calculated the limit of detection value as 20 ng/mL in the range of 20–100 ng/mL. They further investigated the storage stability of the membranes, and the optical sensor could be stable for a year when it was stored at 22 °C. Therefore, the sensor presented a good candidate for food safety screening. Since the possible presence of antibiotics in the environment, the monitoring of water pollutants is globally essential with the emergence of strains resistant to antibiotics. This, therefore, increases the need for the development of portable and cost-effective analytical detection tools for monitoring these substances in water.

**Figure 3:**
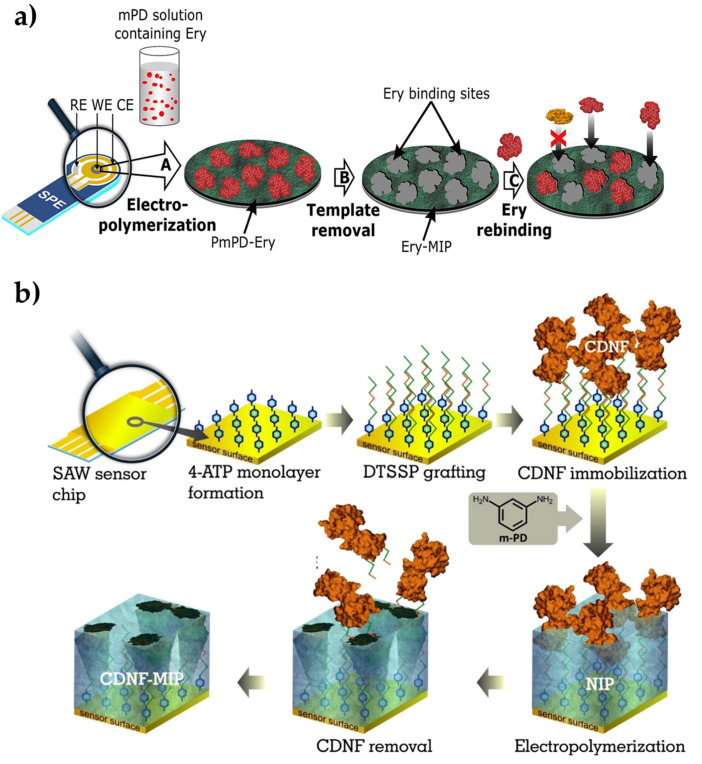
Preparation of the erythromycin-imprinted electrochemical sensor (**a**) and cerebral dopamine neurotrophic factor protein-imprinted surface acoustic wave sensor (**b**). Republished with permission from Ayankojo et al. and Kidakova et al. [87,88].

Ayankojo et al. [87] introduced a methodology that combines a molecularly imprinted polymer (as a sensing element) with a portable electrochemical transducer for environmental monitoring approaches. For this purpose, they prepared an electrochemical sensor for erythromycin detection. The erythromycin-imprinted polymer was first created on-screen imprinted electrode through the polymerization of m-phenylenediamine (Figure 3a). After the optimization process, the kinetic studies were performed in the range of 12.8 nM–40 μM erythromycin concentration. Then, the detection and quantification limits were calculated as 0.1 nM and 0.4 nM, respectively. They tested the electrochemical sensors with the detection of sulfamethizole, amoxicillin, and ciprofloxacin antibiotics, which are not closely related to both buffer and environmental tap water samples, but they are very close analogs to azithromycin and clarithromycin. While they observed strong selectivity for erythromycin against unrelated antibiotics, it showed remarkable discrimination against the close analogs. The electrochemical sensor also presented good recovery in tap water, resulting in 91% to 102%. Overall, in this study, they reported the feasibility of achieving a portable and selective sensor for detecting erythromycin in water by exploiting the synergistic effect of combining the high affinity derivable from imprinted polymer with the compact nature of screen-printed electrode.

Kidakova et al. [88] prepared a surface acoustic wave sensor using molecularly imprinted films to detect cerebral dopamine neurotrophic factor protein. Detecting this protein in the early stages of the disease simplifies the follow-up of neuroprotective therapies. In this study, the researchers utilized an electrochemical surface imprinting method to synthesize a protein-imprinted film and an interface on the sensor (Figure 3b). The optimum thickness of the imprinted layer was then adjusted to increase the recognition capacity and selectivity performance, and they observed that 4.7 nm of thickness was working properly. The selectivity of the sensor has been demonstrated by competitive binding experiments with mesencephalic astrocyte-derived neurotrophic factor. Then, the sensor detected the target protein from 0.1 pg/mL in a wide range (5.0–300 ng/mL). As a preliminary study, this work presented a cost-effective alternative to the current methods in the diagnosis of neurodegenerative diseases. Overall, molecularly imprinted polymers are demonstrated as a highly selective strategy for the investigation of disease-causing factors for public health.

Özgür et al. [89] created a specific recognition receptor using a micro-contact imprinting technique for the detection of urinary tract pathogens (*Escherichia coli).* The optical sensor provided real-time and label-free detection in aqueous and artificial urine solutions within a concentration range of 10^1^ – 10^6^ cfu/mL of *Escherichia coli*. In this study, an amino acid-based monomer was used as a functional monomer to design high selective cavities on the polymeric film of the optical sensor surface. Silver nanoparticles were integrated into the procedure during the preparation of imprinted film, and this enabled an improvement in the limit of detection, achieving down 0.57 cfu/mL within ~20 minutes, which is shorter than conventional bacteria detection methods. The presented optical sensor was designed for the detection of *Escherichia coli* in artificial urine samples that could be potentially applied to detect other biomarkers in urinary infections in the future.

Feng et al. [90] prepared an optosensing platform by using imprinted polymers and quantum dots for the detection of tetrabromobisphenol-A. The imprinted layer was fabricated onto quantum dots using a sol-gel polymerization strategy, hence gaining the sensor fluorescence capability. The characterization studies showed that the composite material had optimal morphological and photoluminescence features. Under the optimized circumstances, high detection linearity was observed in the concentration range of 1.0–60.0 ng/mL. The limit of detection was reported as low as 3.6 ng/g. The fluorescent sensor was used efficaciously for the detection of tetrabromobisphenol-A in the electronic waste samples. Average recoveries were compared with the results of the high-performance liquid chromatography-ultraviolet detection system and they were reported in the range of 89.6% to 107.9% according to this method. Electric fan and circuit board samples were also used to realize real sample studies and the average concentration was found as 260.20 and 707.30 mg/kg. This study demonstrated as an alternative strategy for the detection of pollutants found in electronic wastes by providing high selectivity and short assay time.

Synthetic cannabinoids have become an important public health problem, given their serious abuse and toxic effects. To control the rise in the use of synthetic cannabinoids, sensors with faster and more precise detection fashions will have a great impact on to hurdle this problem. As an example, Akgönüllü et al. [91] developed a piezoelectric sensor**-**coated with imprinted nanoparticles that were prepared through the emulsion polymerization method to detect synthetic cannabinoids. By measuring the mass change due to the binding of synthetic cannabinoids to the sensor surface, it was observed that the sensor could detect as low as 0.28 pg/mL for different cannabinoids in artificial saliva samples, and could provide a high dynamic detection range between 0.0005 ng/mL and 1.0 ng/mL. All the presented work here are also stated in a comparison table (Table 3).

### 4.3. Lab-on-a-Chip Systems

Microfluidic technologies that require a small volume of sample, like only a droplet, offer significant advantages over traditional platforms to detect targets in a short period of assay time [92]. Microfluidics, also known as lab-on-a-chip systems, is the technology that processes small amounts of liquids with channels of tens to hundreds of micrometer sizes [93]. Molecularly imprinted polymers have been successfully integrated with lab-on-a-chip systems for a wide variety of applications [94,95]. Despite the conventional microfluidic strategies, the molecularly imprinted polymers have been leveraging the performance of these systems by increasing chemical reactivity; providing higher surface area; creating specific binding regions to target molecules; increasing the binding capacity; and forming homogeneous spherical geometry [96].

For instance, Wagner et al. [97] combined fluorescent imprinted particles with a droplet-based three-dimensional microfluidic system to selectively identify 2,4-dichlorophenoxyacetic acid in water samples of 20 nM–5.0 μM concentration range. Here, they used a custom-made fluorescent cross-linker to monitor specific binding events through measuring the fluorescent signals. Briefly, the crosslinker was co-polymerized into a target-specific imprinted layer that was attached to the surface of the particles using a reversible chain transfer polymerization (RAFT). They integrated the fluorescent sensor into a modular microfluidic system that permits an in-line phase-transfer assay to extract the analyte from aqueous droplets into the organic phase. The microfluidic system could detect 2,4-dichlorophenoxyacetic acid down to 20 nM. In this study, a fluorescent probe monomer with a cross-linker molecule improved the performance of fluorescent imprinted polymers and the combination of these particles with a microfluidic system could yield a simple yet very powerful miniaturized tool for environmental analysis.

Qi et al. [98] developed an origami ion-imprinted polymer and integrated it with a microfluidic system for the detection of Cu^2+^ ions. In this system, the polymer surface was activated with quantum dots and they achieved the synthesis of ion-imprinted polymers with amine modifications. Since the photoluminescent energy of the quantum dots could be delivered to the prepared complex, fluorescent quenching occurred. The complex of quantum dots and ion-imprinted polymers was transferred to solid glass fiber paper, thereby improving the portability of the device. It has been observed that this developed system shows good linearity for Cu^2+^ between 0.11 to 58.0 μg/L with a detection limit of 0.035 μg/L. Thus, this system can supply quantitative information conveniently and demonstrate a great potential to be further extended to other metal ions detection for environmental monitoring and food safety field.

**Figure 4:**
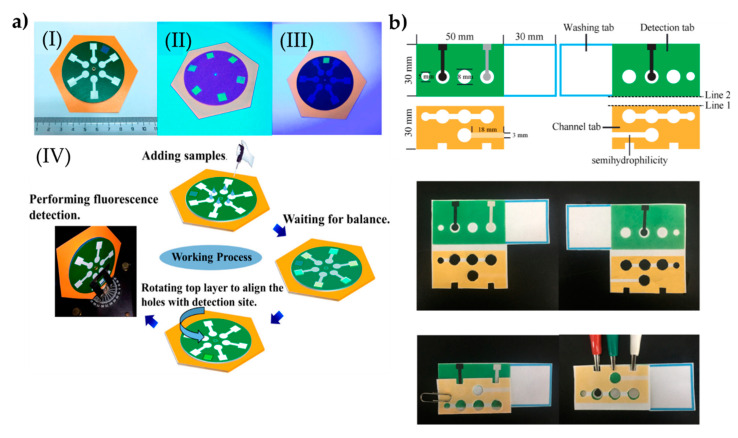
The preparation process of the paper-based microfluidic systems for the determination of phenolic contaminants (**a**). The indicators in the plot: a complete chip under daylight (I), image of the six test sites on the chip under UV light (II), image of the test site through the hole of the top sampling layer under UV light (III), and schematic of the entire working process of the rotational chip and an image of rotational paper-based microfluidic chips placed and the detection process in the fluorescence spectrometer (IV). The preparation steps of the system to determine glycoprotein ovalbumin (**b**). Republished with permission from Qi et al. and Sun et al. [99,100].

The same research group also prepared a rotational paper-based microfluidic system for the detection of phenolic contaminants (Figure 4a). The system was able to provide both qualitative and quantitative analysis of 4-nitrophenol and 2,4,6-trinitrophenol. Determinating these contaminants were analyzed through fluorescent intensity changes. This rotational paper-based microfluidic chip provided an inexpensive, versatile, and easy-to-use approach, and provided high sensitive and selective measurements. In addition, this system detected 4-nitrophenol and 2,4,6-trinitrophenol within a range of 0.5 to 20.0 mg/L and was able to detect them down to 0.097 and 0.071 mg/L, respectively. Overall, the presented platform holds the potential to detect other pollutants for environmental monitoring and food safety [99].

Sun et al. [100] designed an approach by hybridizing chain reaction with imprinted polymers and turned into a paper-based tool for the detection of ovalbumin. The gold nanorod used in the fabrication of the microfluidic tool provided high conductivity on a large surface area through the sandwiching assay strategy. Here, an imprinted polymer was modified with 4-mercaptophenyl boronic acid to successfully capture the ovalbumin. The nanocomposites were labeled with cerium dioxide and then modified with the nicked DNA double-strand polymers, therefore they could detect ovalbumin molecules within the range of 1 pg/mL–1000 ng/mL (Figure 4b). The low detection limit was observed as 0.87 pg/mL. Overall, the presented platform holds pivotal potential in clinical diagnosis and therapeutic monitoring. All the presented work here are also stated in a comparison table (Table 4).

## 5. Conclusions

In this review, we state how natural information could be translated into a biomimetic system by creating molecular fingerprints of host molecules. We also present recent advances in the literature where these molecular fingerprints are decorated on the MIPs and they provide examples in chromatographic systems, microfluidic systems, and sensor systems. Of course, MIP strategy is not only limited to these fields, and their applications have been expanded to regenerative medicine, drug delivery systems, and molecular forensics for which biological imitation has great interest in tackling challenges in biology and medicine. From a biorecognition strategy perspective, in particular, MIP-stemmed systems have some superior advantages over immunospecific systems, including stability, long shelf-life, easy to produce, and low-cost. The materials used in this strategy need to be well-defined according to the process; for instance, rigid polymers might lack flexibility, whereas smoother polymers might easily respond to environmental changes, such as pH and temperature. This point would be improved with the co-polymerization of polymeric materials owing to their distinct features for tuning the product’s mechanic characteristics. On the other hand, MIPs denoted in this review have already been adapted for the recognition of host molecules in biological matrices. These systems, especially in sensor platforms, have one critical drawback, i.e., non-specific binding of other molecules. Although the MIPs are so specific to the host molecules, these molecules mostly form a complex structure with bodily fluids; for instance, drugs are mostly in bound form with serum proteins in circulation. For this manner, the parameters to design the MIPs need to be re-visited for improving surface specificity. Especially, anti-fouling agents could be integrated into the pre-polymer matrix before the generation of MIP structure. Computation-assisted modeling systems/simulations and docking studies would also have a great impact to guide the process and fabricate a more efficient recognition system. These improvements and appropriate updates on the MIP formulation and strategies would expand their usability and applicability larger than ever.

## Figures and Tables

**Table 1:** The comparison of the different polymerization types. Republished with permission from Rutkowska et al. [62]**.**

Polymerization	General Advantages and Disadvantages
Bulk	• Simple and universal type of polymerization. • No need for sophisticated instrumentation. • Obtaining spherical materials. • Providing reproducible results • Allowing a large-scale examination of products. • Requiring lengthy procedures. • Resulting in irregularity in size and shape. • Low performance.
Precipitation	• Providing uniform size and high yields of imprinted materials • Creating homogeneous binding sites. • One of the easiest and well-suited type with a high dilution factor. • Requirement for a polymerization mixture in the presence of a much higher amount of porogen maker. • The growing polymer chains are unable to occupy the entire volume.
Suspension	• An organic-based medium is mixed with an excess of water and the amount of suspension stabilizer. • Two phases are mixed by stirring to form a suspension of organic droplets in the aqueous phase. • The imprinted materials are scarce because water might disrupt non-covalent interactions between the template molecule and the monomers.
Multi-step swelling	• Producing mono-disperse and outstanding materials with controlled diameter. • The size of the imprinted materials might be controlled by changing the polymerization conditions. • Requiring complex and long polymerization conditions. • Requiring laborious procedure and aqueous emulsions.
Surface	• Producing mono-disperse materials and thin imprinted layers. • Creating more accessible binding sites. • Allowing rapid binding and high desorption rates. • Providing more effective ability to recognize the template molecules. • Providing a large specific surface area for the particles, hence leading to excellent affinity and selectivity. • Requireing a complicated system and time-consuming procedure.
In-situ	• Requiring a single-step preparation strategy. • Beinga cost-friendly fashion. • Providing a, well-porous structure. • Requiring a comprehensive and lengthy optimization procedure that needs to be optimized for every template molecules systems.

**Table 2:** Comparison of molecularly imprinted chromatographic systems.

Application	Template Molecule	Polymerization Type	Dynamic Range	Adsorption Capacity	Reference
HPLC-UV	Fluoxetine	Bulk	0–1.5 mM	800 µmol/g	[69]
SDS-PAGE	Concanavalin A	Surface	0–2.0 mg/mL	305.2 mg/g	[70]
NanoLC-UV	Cocaine	In-situ	100–2000 ng/mL	Not available	[71]
HPLC-UV	Quercetin	Sol-gel	0.05–100 μg/mL	19.98 ng/g	[72]
HPLC	Norfloxacin	Precipitation	1.0–200 μg/L	32 mg/g	[73]

**Table 3:** Comparison of molecularly imprinted sensor systems.

Sensor Type	Template Molecule	Polymerization Type	Dynamic Range	Limit of Detection	Reference
Optical	*Enterococcus faecalis*	Emulsion	2 × 10^4^–1 × 10^8^ cfu/mL	1.05 × 10^2^ cfu/mL	[85]
Optical	Aflatoxin B1	In-situ	20–100 ng/mL	20 ng/mL	[86]
Electrochemical	Erythromycin	Electro-polymerization	12.8 nM–40 μM	0.1 nM	[87]
Surface acoustic wave	Cerebral dopamine neurotrophic factor protein	Surface	5.0–300 ng/mL	0.1 pg/mL	[88]
Optical	*Escherichia coli*	Micro-contact	10^1^–10^6^ cfu/mL	0.57 cfu/mL	[89]
Fluorescent	Tetrabromobisphenol-A	Sol-gel	1.0–60 ng/mL	3.6 ng/g	[90]
Piezoelectric	Cannabinoids	Emulsion	0.0005–1.0 ng/mL	0.28 ng/mL	[91]

**Table 4:** Comparison of molecularly imprinted lab-on-a-chip systems.

Combination	Template Molecule	Polymerization Type	Dynamic Range	Limit of Detection	Reference
Fluorescent sensor	2,4-dichloro phenoxyacetic acid	RAFT	20 nM–5 μM	20 nM	[97]
Fluorescent sensor	Cu^2+^	Surface	0.11–58 μg/L	0.035 μg/L	[98]
Fluorescent sensor	4-nitrophenol	Surface	0.5–20 mg/L	0.097 mg/L	[99]
Electrochemical sensor	Ovalbumin	In-situ	1 pg/mL–1000 ng/mL	0.87 pg/mL	[100]
